# A mixed‐methods approach to understand victimization discourses by opposing feminist sub‐groups on social media

**DOI:** 10.1111/bjso.12785

**Published:** 2024-07-05

**Authors:** Christina Maxwell, Hema Preya Selvanathan, Sam Hames, Charlie R. Crimston, Jolanda Jetten

**Affiliations:** ^1^ The University of Queensland Brisbane Queensland Australia; ^2^ The Australian National University Canberra Australian Capital Territory Australia

**Keywords:** collective victimhood, critical discursive psychology, feminism, inclusion, social media, social movements, topic modelling

## Abstract

Opposing social movements are groups that have conflicting objectives on a shared social justice issue. To maximize the probability of their movement's success, groups can strategically portray their group in a favourable manner while discrediting their opposition. One such approach involves the construction of victimization discourses. In this research, we combined topic modelling and critical discursive psychology to explore how opposing groups within the feminist movement used victimization as a lens to understand their movements in relation to transgender women. We compiled a dataset of over 40,000 tweets from 14 UK‐based feminist accounts that included transgender women as women (the pro‐inclusion group) and 13 accounts, that excluded transgender women (the anti‐inclusion group). Our results revealed differences in how victimization was employed by the opposing movements: pro‐inclusion groups drew on repertoires that created a sense of shared victimhood between cisgender women and transgender women, while anti‐inclusion groups invoked a competitive victimhood repertoire. Both groups also challenged and delegitimised their oppositions' constructions of feminism and victimhood. These findings add to our understanding of the communication strategies used by opposing movements to achieve their mobilization goals.

## BACKGROUND

Attempts to incite social change seldom occur without opposition. Consequently, social movement groups with differing agendas on an issue can find themselves competing to obtain resources and elicit public and political support to increase the success of their mobilization objectives (Thomas & Osborne, 2022). To gain a competitive advantage, groups strategically craft their movement communication in ways that positively distinguish the ingroup from their opposition. As social movements are propelled by a narrative of oppression, these lines of distinction often position the ingroup as victims and the outgroup as the perpetrators of their victimization (Bar‐Tal et al., 2009).

Feminism is a social movement that often utilizes a victimization narrative to advocate for gender‐based justice. However, feminism is not a homogenous movement, and the designation of victim and perpetrator identities is contested among its various sub‐groups. For instance, in recent years there has been considerable tension regarding the role of transgender (trans) women in the feminist movement. One sub‐group, consisting of individuals labelled as trans‐exclusionary radical feminists (TERFs) by others such as JK Rowling or self‐defined ‘gender critical feminists’, seeks to remove trans individuals from feminist organizing by creating distinctions between ‘trans issues’ and ‘women's issues’ (Green, 2008; Shaw, 2023). Alternatively, other feminists consider that trans‐inclusionary practices are not only consistent with feminist ideology but are also necessary for the movement's success (Kirkland, 2019; Williams, 2016; Withers, 2010).

While both movements aim to improve the conditions of ‘women’ (however ‘women’ are defined), the goals and the actions needed to achieve them differ between groups and can directly conflict with each other. The current research compares how opposing groups within the feminist movement frame their movements using victimization discourses regarding the inclusion of trans women.

## OPPOSING MOVEMENTS IN FEMINISM

Social Identity Theory (SIT; Tajfel & Turner, 1979) posits that groups obtain a positive identity from favourable comparisons with relevant outgroups. In response to perceptions of injustice against one's ingroup, group members may attempt to change their social conditions through collective action. The success of social movements requires the mobilization of disparate individuals into a singular entity that shares a sense of identity and movement goals.

However, social movements do not exist in a vacuum; issues attract multiple interests with conflicting goals such that groups must often compete for scarce public attention, support, and resources (Thomas & Osborne, 2022). These opposing groups may have distinct identities, or they may constitute sub‐groups within a larger superordinate group. For instance, different Aboriginal and Torres Strait Islanders communities in Australia held contrasting perspectives and objectives regarding the recent 2023 Voice to Parliament referendum, a proposed change to the Australian Constitution to recognize the First Peoples of Australia by establishing a representative advisory body to the Parliament. One sub‐group of Aboriginal and Torres Strait Islander community members believed that the Voice undermined their Sovereignty and advocated for a ‘No’ vote in the referendum while another sub‐group campaigned for a ‘Yes’ vote by recognizing the Voice as an important step forward in First Nations' justice (Blak Sovereign Movement, 2023; Gerrard, 2023).

Understanding these sub‐groups in context is important because the success of a given social movement requires the consideration of opposing groups. Despite the importance of this comparative lens, social psychological research that examines the communication strategies of and dynamics between opposing movements remains in its infancy (for existing research, see Brown et al., 2022; Milesi & Alberici, 2018; Selvanathan et al., 2021; Solomon & Martin, 2019). Recent calls from Thomas and Osborne (2022) have sought to increase the literature on opposing groups, arguing that one‐sided perspectives do not adequately reflect the social movement experience. Social change is characterized by push‐and‐pull relationships of competing groups, necessitating a holistic understanding of the various perspectives that co‐exist. In the current work, we address these calls through an examination of the feminist movement.

In general, feminism is built on the idea that there are unfavourable group comparisons between women and men, such that the former are socially and legally disadvantaged as compared to the latter. Feminism is not an uncontested term, however, and it has been defined in different ways by various sub‐groups regarding the goals of the movement and the ways to achieve them. One issue across which feminism is split regards trans issues, most notably whether trans women should be included within the boundaries of ‘womanhood’ and therefore included in movement goals to emancipate women from patriarchal structures (Shaw, 2023). On the one hand, there is a majority sub‐group of feminists who view trans women as women and advocates to advance the rights of both trans women and cisgender (cis) women (Williams, 2016). Another sub‐group positions trans women as outgroup members and therefore distinct from feminist organizing (Green, 2008). In this research, these two groups are referred to as pro‐inclusion feminists and anti‐inclusion feminists, respectively. These two opposing groups can also be distinguished by their endorsement of gender essentialism, which is the belief that men and women represent diametrically opposed and distinct categories determined by biological characteristics which in turn determines norms around behaviour and societal roles (Morgenroth & Ryan, 2021). This perspective is binary in nature, thereby rejecting transgender and gender‐diverse identities and underpins the boundary maintenance strategies of anti‐inclusion feminists (Armitage, 2020; Hines, 2020).

## ORGANIZING THE ONLINE SOCIAL MOVEMENT

Social media is becoming an increasingly important medium for social movement organizing (e.g., Adam‐Troian et al., 2021; Odaǧ et al., 2016). Platforms such as Twitter (now X) are often used by activists to build movement momentum by dispersing information to a global audience, connecting like‐minded people, and coordinating collective action both online and offline. Feminist movements also utilize these benefits of social media organizing (Li et al., 2021; Storer & Rodriguez, 2020; Willem et al., 2022). Consequently, these qualities make Twitter an ideal data source to observe how opposing feminist sub‐groups communicate in a naturalistic setting.

To‐date, research on the intersections between feminism and the trans community has largely focused on documenting anti‐inclusion perspectives, including the detection of these accounts and the understanding of their anti‐trans hate speech (Lu & Jurgens, 2022; Quatrini, 2022; Willem et al., 2022). Feminists with pro‐inclusion attitudes have often been eclipsed by this focus on anti‐inclusion feminists in both news media and research. By comparing the two opposing movements, we can not only understand the ways in which each group differentially constructs the feminist movement online, but we can also document the under‐investigated views of pro‐inclusion feminists. The next section overviews victimization discourses as one way in which opposing groups may construct their movements to increase the success of their mobilization objectives.

## VICTIMIZATION DISCOURSES

Self‐perceived collective victimization (Bar‐Tal et al., 2009; Vollhardt, 2020) is central to social movement organizing as it signals an undeserved injustice towards one group at the hands of another group. It functions as a shared group‐based perception where ingroup members believe (accurately or not) that they have been victimized by an outgroup. Victimization provides a lens through which group members can understand their current group conditions, motivates mobilization, justifies actions towards the outgroup, and offers a manifesto for bystanders that inspires sympathy and investment in the group's objectives (Benford & Snow, 2000).

As such, ingroups create positive inter‐group distinctions by categorizing themselves as vulnerable victims and outgroups as amoral perpetrators who are subsequently dehumanized and delegitimised (Smith et al., 2012). Far from being an undesirable identity signalling weakness, victimized identities can be used by groups to gain collective esteem by claiming a position of moral superiority as compared to perpetrator groups (Bar‐Tal et al., 2009; Mijić, 2021). Two distinct collective victimhood processes have been identified: inclusive victim consciousness and exclusive victim consciousness (Vollhardt, 2015).

An inclusive victim consciousness acknowledges the suffering and victimization status of outgroups alongside that experienced by one's own ingroup (Vollhardt, 2015). Often, this framing is facilitated when other groups are victimized by the same perpetrators as the ingroup (Craig & Richeson, 2012; Vollhardt, 2015). Groups that adopt this mindset may make connections between their own victimization and that of other groups and acknowledge that the fates of the two groups are linked (Dawson, 1994). Consequently, collaborative action is perceived as mutually beneficial and ingroup boundaries are expanded to accommodate other groups. However, some groups may reject this perspective in preference of one that maximizes their distinctiveness from other groups.

Groups that adopt an exclusive victim consciousness see their suffering as unparalleled (Vollhardt, 2015). Consistent with SIT (Tajfel & Turner, 1979), to the degree that adopting a victimized identity is favourable for collective esteem, groups may attempt to maximize their differences from others by claiming to be the *most* victimized. This competitive victimhood (Noor et al., 2012) results in the downplaying or outright dismissal of other groups' suffering while the suffering of the ingroup is emphasized. Group members who use this framing often engage in zero‐sum thinking by believing that the advancement of another group on a shared dimension of interest comes at the direct cost of their own group's advancement (Norton & Sommers, 2011).

Collective victimization and competitive victimhood theories have typically been applied to understanding protracted intergroup conflict at a national level (Szabó, 2020). However, Solomon and Martin (2019) have argued that these frameworks can be relevant to social movement settings, as demonstrated through their application of competitive victimhood to the Black Lives Matter and Blue Lives Matter (a counter‐movement focused on police rights) movements in the United States. In this work, we propose that opposing groups within the feminist movement use victimization discourses in their communication around trans women, albeit in different ways. For instance, to the degree that trans women are positioned as politically, socially, and legally similar to cis women, these groups may experience feelings of similarity (resulting in an inclusive victim consciousness) or threat (resulting in an exclusive victim consciousness). As such, in the current research we use a victimization lens and the broader social identity theory framework (Turner & Reynolds, 2001) to compare how these opposing groups discursively construct their feminist movements in relation to trans women.

## THE CURRENT RESEARCH

To address our aim, we used a mixed‐methods approach combining topic modelling with critical discursive psychology using Twitter data. Topic modelling served two purposes: to provide a broad overview of each group's primary concerns for their mobilization and to reduce the dataset for more detailed, qualitative analysis. The breadth of social media data available makes it time‐consuming and difficult to detect patterns by hand. Computer‐assisted methods of automated text analysis can help to extract major topics and provide a starting point for further qualitative analysis (Baker et al., 2008). Specifically, we used latent dirichlet allocation (LDA) topic modelling (Blei et al., 2003) which is an unsupervised machine learning method that separates texts into individual words, detects patterns in word usage, and clusters together words that frequently co‐occur together into topics. Each document (i.e., tweet) is reduced to a collection of topics that capture the key themes discussed and provide a broad summary of the content. We used these topics as a proxy for the dimensions on which opposing groups consider themselves to be victimized and therefore the focus of their feminist movement goals.

We then analysed the results of the topic model using critical discursive psychology to explore how these opposing groups employed victimization frameworks to position themselves, their movements, and opposing groups with regards to trans women. Critical discursive psychology is concerned with how language is used to construct versions of reality to serve specific purposes within a specific socio‐cultural context at a particular moment in time (Edley, 2001; Potter & Wetherell, 1987; Wiggins, 2017). As aforementioned, employing victimization discourses can be strategically advantageous for groups in conflict to serve various goals.

Together, this mixed‐methods approach allows us to identify the global patterns that distinguish these two movements in their mobilization focus (via topic modelling) as well as how these different movements are constructed at the granular level of discourse (via critical discursive psychology). This sequential approach to analysing social media data contributes to a rigorous and holistic research process (Isoaho et al., 2019) and has been used by other social psychology researchers (Goodman et al., 2024; Shayegh et al., 2023; Törnberg & Törnberg, 2016; Windel et al., 2022). It is important to note that while these methods have only recently emerged in social psychology, critical discourse scholars have long‐since recognized how corpus linguistics and discourse analysis can complement and strengthen each other (Baker, 2012; Baker et al., 2008; Gabrielatos et al., 2012).

We applied these methods to a dataset consisting of opposing feminist groups in the UK. The UK was selected due to the division between these sub‐groups over recent years (Armitage, 2020; Hines, 2020; Withers, 2010). For example, the recent passing of the Gender Recognition Reform Bill in Scotland (a Bill that intended to ease the process by which trans individuals could have their gender identity legally recognized; Smout & MacAskill, 2023) and the subsequent blocking of the Bill in the UK resulted in a considerable amount of public discourse, particularly among feminists. Subsequently, pro‐inclusion and anti‐inclusion groups have advocated for policy that aligns with their respective goals.

## METHODS

### Data

We manually examined Twitter for UK‐based anti‐inclusion and pro‐inclusion accounts. This sampling strategy was supplemented with Twitter search tools^1^ in which various search terms and hashtags were entered (e.g., ‘feminist’, ‘trans‐inclusionary’, #sexnotgender, #savewomensports, #adulthumanfemale, London, Scotland) and relevant users were returned. Accounts were deemed eligible if they: were public, were geographically tagged as being in the UK, focused on advancing women's rights, did not represent personal individual accounts, and made a statement about whether trans women are included in their definition of ‘women’. We excluded accounts that were minimally active during the data collection period, did not post original content (i.e., accounts that only re‐posted others' content), and were local nodes of larger national groups as we observed a high degree of retweeting and low original content in the former. This strategy resulted in 13 anti‐inclusion accounts and 14 pro‐inclusion accounts. An anonymized overview of these accounts is provided in the Data S1 section.

Data were scraped from the Twitter API using the Python library *twarc* (Summers et al., 2023) from January 2020 through to March 2023. This period was selected to capture the time around which anti‐inclusion perspectives were becoming increasingly vocal on Twitter, driven in part by JK Rowling (see Gardner (2023) for an overview) as well as the discussions surrounding the Gender Recognition Act. A total of 32,608 tweets were collected for the anti‐inclusion accounts and 11,248 tweets for the pro‐inclusion accounts.

### Topic modelling

First, the dataset was cleaned for analysis by removing URLs, non‐English characters, text that appeared within quotations, punctuation, numbers, and stopwords (common words that do not provide substantive information about a topic; additional detail about data cleaning processes can be found in the Data S1). The final dataset included 10,545 pro‐inclusion tweets and 30,128 anti‐inclusion tweets.

The topic models were computed in R using the *topicmodels* package (Grün & Hornik, 2011, 2024, version 0.2‐16). The number of topics (designated by *k*) must be determined by the user prior to running the topic model, although there is disagreement in the literature regarding the best approach (Bystrov et al., 2022). We combined statistical techniques with subjective interpretation (Grimmer & Stewart, 2013; Isoaho et al., 2019) to identify the best‐fitting number of topics based on close‐reading of the most representative tweets. ‘Best fit’ was determined by a topic structure that delivered distinct, independent topics that were representative of the dataset and addressed the research aims (Giorgi et al., 2022). The first author computed cumulative sets of 5 topic models from 5 through to 55 (this upper cut‐off was determined by the evaluation metrics available in the *ldatuning* package in R; Nikitia, 2020) and manually examined the top 20 terms and top 20 tweets in each topic for best fit in consultation with the third author. Through this process, we decided on 15 topics for the anti‐inclusion group and 20 topics for the pro‐inclusion group.

### Critical discursive psychology

To understand how each opposing movement utilized victimization discourses in their communication, we identified the topic in each group that contained the strongest trans‐focus (as determined by assessing *beta* of the term ‘trans’ on a given topic). The tweets in each topic were then analysed using critical discursive psychology, guided by other researchers' applications of this methodology (Edley, 2001; Locke & Budds, 2020).

To start, we repeatedly reviewed both datasets and noted instances where victimization was invoked. We used a partially deductive approach to identify discourse indicative of inclusive and exclusive victim consciousness while remaining open to other ways of discussing victimization. We then observed how patterns informed the development of interpretative repertoires, which are the lenses that are used to make sense of an issue (Edley, 2001; Potter & Wetherell, 1987). At this stage, we also considered the ways in which these repertoires were related to each other, resisted by the opposing groups, and employed to accomplish specific goals. Within these repertoires, we identified the subject positions, that is, the identities and their associated attributes that are assigned to the ingroup and outgroup (Locke & Budds, 2020). Finally, we examined how these subject positions and interpretative repertoires were constructed by identifying the discursive devices used and their intended functions with reference to the broader socio‐political context.

To comply with Twitter's Terms and Conditions and users' expectations of how their data are used (Fiesler & Proferes, 2018), we do not reproduce tweets in full to limit re‐identifiability. No consent was sought for any of the included accounts; we argue alongside other researchers (e.g., Kychenthal, 2022) that public activist groups on Twitter intend for their posts to be widely accessible to meet their objectives of social change via public awareness‐raising. The current project also follows the Association of Internet Research Ethics Guidelines 3.0 (Franzke et al., 2020).

## RESULTS

### Topic modelling

The complete results of the topic models are presented in Tables 1 (pro‐inclusion) and 2 (anti‐inclusion). Here, we provide a summary of the topics relevant to our aim of identifying the mobilization objectives of each movement. Pro‐inclusion groups are mobilized towards a range of issues such as sex work (Topic 2), support for women during COVID‐19 (Topic 4), reproductive rights (Topic 6), the trans community (Topic 11), police violence towards women (Topic 15), domestic violence (Topic 16), and public sexual harassment (Topic 18). As would be expected for feminist‐aligned groups, two topics are dedicated to calls to action for their agendas (Topic 9 and Topic 13).

**TABLE 1:** Results of LDA topic modelling for pro‐inclusion accounts.

Topic	Top 20 terms (beta)
1	**Will** (0.127)**; take** (0.036)**; make** (0.034)**; year** (0.030)**; time** (0.026)**; now** (0.026)**; back** (0.024)**; many** (0.024)**; keep** (0.021)**; look** (0.017)**; may** (0.016)**; ask** (0.013)**; getting** (0.013)**; way** (0.010)**; wont** (0.010)**; nothing** (0.010)**; sure** (0.009)**; old** (0.009)**; people** (0.008)**; friends** (0.008)
2	**Sex** (0.076)**; work** (0.051)**; workers** (0.043)**; end** (0.027); **prison** (0.025)**; pregnant** (0.023)**; justice** (0.020)**; safety** (0.015)**; years** (0.015)**; demand** (0.013)**; fight** (0.013)**; poverty** (0.012)**; outside** (0.012)**; baby** (0.010)**; ecp** (0.007)**; laws** (0.007)**; dangerous** (0.006)**; stop** (0.006)**; speak** (0.006)**; babies** (0.006)
3	**Purpleheart** (0.076)**; love** (0.042)**; thank** (0.033)**; feminist** (0.033)**; raisedfist** (0.031)**; solidarity** (0.028)**; community** (0.025)**; much** (0.023)**; redheart** (0.021)**; show** (0.015)**; incredible** (0.015)**; transallyscot** (0.014)**; stand** (0.013)**; link** (0.012)**; full** (0.011)**; always** (0.010)**; beautiful** (0.009)**; scotland** (0.009)**; art** (0.009)**; sharing** (0.009)
4	**Women** (0.210)**; support** (0.094)**; working** (0.022)**; covid** (0.018)**; services** (0.014)**; money** (0.013)**; single** (0.013)**; access** (0.012)**; including** (0.011)**; living** (0.010)**; crisis** (0.009)**; parents** (0.009)**; issue** (0.009)**; currently** (0.008)**; womens** (0.007)**; network** (0.007)**; issues** (0.007)**; asylum** (0.006)**; cost** (0.006)**; costs** (0.006)
5	**Day** (0.065)**; new** (0.064)**; today** (0.048)**; read** (0.038)**; great** (0.035)**; womens** (0.035)**; happy** (0.025)**; time** (0.025)**; check** (0.023)**; weve** (0.022)**; every** (0.021)**; international** (0.014)**; backhandindexpointingdown** (0.013)**; post** (0.012)**; article** (0.009)**; celebrate** (0.009)**; together** (0.008)**; blog** (0.008)**; around** (0.008)**; iwd** (0.007)
6	**Rights** (0.069)**; right** (0.056)**; government** (0.021)**; abortion** (0.020)**; womens** (0.020)**; human** (0.017)**; groups** (0.012)**; reproductive** (0.012)**; needs** (0.011)**; trying** (0.011)**; must** (0.010)**; far** (0.010)**; culture** (0.010)**; now** (0.009)**; claim** (0.008)**; based** (0.008)**; bodies** (0.008)**; people** (0.008)**; members** (0.008)**; attack** (0.007)
7	**Keycap** (0.060)**; free** (0.053)**; join** (0.037)**; come** (0.024)**; online** (0.023)**; find** (0.021)**; centre** (0.020)**; next** (0.018)**; info** (0.014)**; tomorrow** (0.013)**; register** (0.013)**; happening** (0.012)**; learn** (0.011)**; along** (0.010)**; course** (0.010)**; sparkles** (0.010)**; group** (0.010)**; welcome** (0.009)**; lots** (0.008)**; whiteflag** (0.008)
8	**Nottingham** (0.029)**; find** (0.023)**; event** (0.023)**; join** (0.023)**; looking** (0.020)**; hear** (0.019)**; apply** (0.014)**; education** (0.013)**; supporting** (0.012)**; events** (0.012)**; team** (0.011)**; small** (0.011)**; eyes** (0.011)**; wed** (0.010)**; work** (0.010)**; fantastic** (0.009)**; organizations** (0.009)**; march** (0.009)**; group** (0.009)**; gea** (0.009)
9	**Also** (0.040)**; sign** (0.033)**; black** (0.029)**; made** (0.024)**; never** (0.024)**; lives** (0.023)**; campaign** (0.019)**; part** (0.015)**; petition** (0.014)**; times** (0.012)**; white** (0.012)**; anything** (0.011)**; using** (0.010)**; tweet** (0.010)**; going** (0.010)**; ever** (0.010)**; women** (0.009)**; weve** (0.009)**; matter** (0.008)**; letter** (0.007)
10	**Don't** (0.084)**; like** (0.066)**; just** (0.064)**; want** (0.063)**; know** (0.054)**; say** (0.038)**; cant** (0.029)**; really** (0.026)**; someone** (0.022)**; think** (0.018)**; tell** (0.015)**; thing** (0.014)**; might** (0.014)**; world** (0.014)**; let** (0.013)**; didn't** (0.009)**; around** (0.009)**; you're** (0.007)**; actually** (0.006)**; almost** (0.006)
11	**Women** (0.124)**; *trans*** (0.120)**; people** (0.059)**; men** (0.051)**; cis** (0.026)**; feminism** (0.021)**; anti** (0.019)**; they're** (0.017)**; movement** (0.017)**; spaces** (0.013)**; inclusive** (0.012)**; lesbian** (0.011)**; feminists** (0.011)**; lesbians** (0.011)**; likely** (0.010)**; youre** (0.010)**; that's** (0.009)**; think** (0.009)**; believe** (0.008)**; anyone** (0.008)
12	**See** (0.064)**; well** (0.053)**; good** (0.041)**; everyone** (0.027)**; thanks** (0.023)**; clappinghands** (0.019)**; yes** (0.018)**; done** (0.013)**; reading** (0.013)**; twitter** (0.011)**; keen** (0.010)**; morning** (0.010)**; live** (0.010)**; instagram** (0.010)**; lets** (0.010)**; wonderful** (0.010)**; hope** (0.009)**; lovely** (0.009)**; jay** (0.008)**; got** (0.008)
13	**Can** (0.122)**; please** (0.068)**; get** (0.048)**; need** (0.041)**; help** (0.040)**; now** (0.033)**; still** (0.032)**; share** (0.030)**; law** (0.015)**; hate** (0.013)**; crime** (0.012)**; social** (0.011)**; legal** (0.010)**; donate** (0.009)**; call** (0.009)**; response** (0.008)**; left** (0.008)**; misogyny** (0.008)**; consultation** (0.006)**; fund** (0.006)
14	**Week** (0.031)**; last** (0.030)**; going** (0.030)**; shop** (0.029)**; open** (0.028)**; get** (0.024)**; email** (0.022)**; bookshop** (0.019)**; two** (0.018)**; pandemic** (0.015)**; online** (0.015)**; days** (0.012)**; business** (0.012)**; tomorrow** (0.011)**; website** (0.011)**; now** (0.011)**; taking** (0.011)**; send** (0.011)**; orders** (0.011)**; since** (0.009)
15	**Police** (0.080)**; fire** (0.029)**; violence** (0.023)**; bill** (0.020)**; protest** (0.020)**; killthebill** (0.016)**; policecarlight** (0.016)**; power** (0.015)**; state** (0.015)**; join** (0.015)**; protect** (0.015)**; collision** (0.013)**; parliament** (0.012)**; london** (0.012)**; action** (0.012)**; powers** (0.011)**; tonight** (0.011)**; racist** (0.009)**; loudspeaker** (0.009)**; met** (0.009)
16	**Violence** (0.055)**; children** (0.041)**; abuse** (0.038)**; domestic** (0.030)**; rape** (0.030)**; family** (0.026)**; stop** (0.023)**; report** (0.022)**; victims** (0.021)**; violent** (0.018)**; court** (0.018)**; must** (0.017)**; courts** (0.016)**; mums** (0.014)**; kids** (0.014)**; mothers** (0.013)**; child** (0.013)**; resources** (0.010)**; survivors** (0.009)**; away** (0.008)
17	**One** (0.079)**; woman** (0.058)**; important** (0.028)**; man** (0.026)**; smilingfacewithheart.eyes** (0.023)**; news** (0.022)**; home** (0.021)**; another** (0.020)**; downarrow** (0.018)**; many** (0.015)**; making** (0.014)**; night** (0.011)**; yet** (0.010)**; office** (0.010)**; just** (0.009)**; especially** (0.009)**; talking** (0.009)**; country** (0.008)**; person** (0.007)**; given** (0.007)
18	**Harassment** (0.053)**; sexual** (0.051)**; public** (0.051)**; girls** (0.034)**; safe** (0.031)**; feel** (0.023)**; change** (0.022)**; need** (0.017)**; campaign** (0.017)**; psh** (0.014)**; young** (0.013)**; street** (0.013)**; health** (0.013)**; streets** (0.013)**; experience** (0.013)**; space** (0.012)**; deserve** (0.012)**; assault** (0.011)**; criminal** (0.011)**; schools** (0.010)
19	**People** (0.069)**; gender** (0.034)**; isnt** (0.022)**; care** (0.021)**; doesnt** (0.020)**; way** (0.020)**; even** (0.018)**; theres** (0.017)**; without** (0.017)**; anyone** (0.014)**; arent** (0.013)**; pay** (0.012)**; saying** (0.011)**; something** (0.011)**; apparently** (0.011)**; thats** (0.010)**; means** (0.010)**; equality** (0.010)**; act** (0.009)**; able** (0.009)
20	**Book** (0.071)**; books** (0.056)**; first** (0.054)**; today** (0.035)**; rare** (0.021)**; sold** (0.021)**; things** (0.017)**; list** (0.016)**; edition** (0.014)**; sell** (0.012)**; writers** (0.011)**; bookstore** (0.010)**; signed** (0.010)**; wonderful** (0.010)**; birthday** (0.010)**; little** (0.010)**; fair** (0.010)**; second** (0.009)**; copy** (0.009)**; put** (0.008)

*Note*: Top terms are presented in descending order by beta (indicated in parentheses), which indicates how strongly a term is related to a particular topic.

**TABLE 2:** Results of LDA topic modelling for anti‐inclusion accounts.

Topic	Top 20 terms
1	**Sex** (0.088)**; gender** (0.051)**; law** (0.028)**; identity** (0.019)**; legal** (0.019)**; can** (0.018)**; act** (0.017)**; census** (0.016)**; change** (0.015)**; equality** (0.014)**; use** (0.014)**; clear** (0.011)**; question** (0.011)**; data** (0.010)**; public** (0.010)**; activists** (0.010)**; court** (0.010)**; anyone** (0.010)**; single** (0.009)**; must** (0.009)
2	**Women** (0.144)**; rights** (0.093)**; womens** (0.076)**; womenwontwheesht** (0.035)**; human** (0.016)**; stand** (0.016)**; solidarity** (0.015)**; day** (0.014)**; every** (0.013)**; iwd** (0.013)**; sexmatters** (0.012)**; since** (0.012)**; welcome** (0.010)**; standing** (0.008)**; wpukicons** (0.008)**; happy** (0.007)**; followers** (0.007)**; sex.based** (0.007)**; sisters** (0.007)**; brave** (0.007)
3	**Women** (0.112)**; men** (0.053)**; violence** (0.026)**; abuse** (0.021)**; man** (0.019)**; hate** (0.018)**; police** (0.016)**; crime** (0.016)**; woman** (0.016)**; rape** (0.015)**; sexual** (0.014)**; misogyny** (0.011)**; apparently** (0.010)**; threats** (0.009)**; violent** (0.009)**; real** (0.008)**; rollingonthefloorlaughing** (0.008)**; victims** (0.007)**; breaking** (0.007)**; thinks** (0.007)
4	**Like** (0.034)**; people** (0.027)**; also** (0.022)**; gov** (0.018)**; really** (0.015)**; group** (0.015)**; groups** (0.015)**; may** (0.013)**; says** (0.012)**; stonewall** (0.011)**; seems** (0.011)**; point** (0.011)**; yet** (0.010)**; rather** (0.010)**; understand** (0.010)**; trying** (0.009)**; believe** (0.009)**; claim** (0.009)**; used** (0.009)**; making** (0.008)
5	**Now** (0.063)**; letwomenspeak** (0.026)**; backhandindexpointingdown** (0.023)**; kellie. jay** (0.023)**; new** (0.019)**; live** (0.019)**; theposieparker** (0.017)**; watch** (0.015)**; get** (0.014)**; full** (0.011)**; going** (0.011)**; love** (0.010)**; link** (0.010)**; look** (0.010)**; twitter** (0.010)**; speaking** (0.009)**; coming** (0.008)**; find** (0.007)**; heres** (0.007)**; can** (0.007)
6	**Thread** (0.062)**; good** (0.040)**; see** (0.040)**; well** (0.036)**; read** (0.027)**; great** (0.024)**; important** (0.023)**; another** (0.023)**; hope** (0.019)**; today** (0.019)**; article** (0.018)**; done** (0.017)**; excellent** (0.016)**; news** (0.016)**; case** (0.016)**; really** (0.015)**; thank** (0.013)**; issue** (0.011)**; comments** (0.008)**; morning** (0.008)
7	**Women** (0.047)**; female** (0.044)**; male** (0.038)**; womens** (0.036)**; *trans*** (0.027)**; girls** (0.020)**; prison** (0.019)**; policy** (0.017)**; sport** (0.016)**; spaces** (0.015)**; males** (0.012)**; prisons** (0.012)**; sports** (0.012)**; services** (0.011)**; safety** (0.011)**; must** (0.010)**; fair** (0.009)**; play** (0.008)**; safe** (0.008)**; single.sex** (0.088)
8	**People** (0.050)**; dont** (0.037)**; know** (0.037)**; just** (0.036)**; think** (0.028); **say** (0.028)**; want** (0.022)**; even** (0.020)**; like** (0.015)**; cant** (0.015)**; never** (0.014)**; tell** (0.014)**; doesnt** (0.012)**; might** (0.012)**; isnt** (0.011)**; wrong** (0.011)**; thats** (0.011)**; wont** (0.010)**; enough** (0.010)**; things** (0.010)
9	**Many** (0.034)**; children** (0.033)**; young** (0.015)**; concerns** (0.013)**; care** (0.012)**; without** (0.011)**; also** (0.011)**; child** (0.011)**; needs** (0.010)**; health** (0.010)**; social** (0.009)**; medical** (0.009)**; issues** (0.009)**; school** (0.009)**; schools** (0.008)**; vulnerable** (0.007)**; others** (0.007)**; parents** (0.007)**; kids** (0.006)**; nhs** (0.006)
10	**Will** (0.021)**; place** (0.018)**; event** (0.017)**; next** (0.016)**; letter** (0.016)**; meeting** (0.015)**; womans** (0.014)**; tickets** (0.013)**; thank** (0.012)**; book** (0.011)**; join** (0.011)**; tonight** (0.010)**; looking** (0.009)**; forward** (0.009)**; online** (0.009)**; open** (0.009)**; week** (0.008)**; march** (0.008)**; tomorrow** (0.007)**; discussion** (0.007)
11	**Can** (0.054)**; please** (0.050)**; support** (0.040)**; will** (0.039)**; thank** (0.034)**; work** (0.025)**; make** (0.024)**; help** (0.022)**; girls** (0.016)**; today** (0.015)**; fight** (0.013)**; share** (0.013)**; action** (0.013)**; wolf** (0.012)**; sexnotgender** (0.011)**; ask** (0.011)**; keep** (0.010)**; sure** (0.010)**; protect** (0.009)**; amazing** (0.009)
12	**Will** (0.039)**; scottish** (0.038)**; scotland** (0.035)**; bill** (0.026)**; said** (0.021)**; evidence** (0.016)**; gra** (0.016)**; notoselfid** (0.015)**; government** (0.013)**; msps** (0.013)**; reform** (0.012)**; party** (0.011)**; consultation** (0.011)**; response** (0.011)**; impact** (0.010)**; questions** (0.010)**; statement** (0.009)**; committee** (0.009)**; guidance** (0.009)**; self** (0.009)
13	**One** (0.056)**; back** (0.023)**; last** (0.022)**; feminist** (0.022)**; year** (0.018)**; time** (0.018)**; everyone** (0.017)**; new** (0.016)**; years** (0.015)**; made** (0.013)**; campaign** (0.012)**; day** (0.012)**; two** (0.011)**; feminism** (0.011)**; part** (0.011)**; education** (0.009)**; edwomenslib** (0.009)**; weve** (0.009)**; justice** ()**; thought** ()
14	**Need** (0.039)**; right** (0.029)**; still** (0.023)**; get** (0.020)**; take** (0.020)**; way** (0.019)**; much** (0.019)**; time** (0.017)**; speech** (0.016)**; political** (0.016)**; speak** (0.014)**; come** (0.013)**; stop** (0.012)**; debate** (0.012)**; women** (0.012)**; better** (0.011)**; feminists** (0.011)**; long** (0.010)**; talk** (0.010)**; free** (0.010)
15	**Woman** (0.032)**; first** (0.029)**; world** (0.017)**; clappinghands** (0.010)**; best** (0.010)**; set** (0.009)**; black** (0.009)**; society** (0.009)**; national** (0.008)**; working** (0.008)**; history** (0.007)**; member** (0.007)**; civil** (0.007)**; activist** (0.007)**; including** (0.007)**; movement** (0.007)**; international** (0.006)**; age** (0.006)**; won** (0.006)**; known** (0.006)

*Note*: Top terms are presented in descending order by beta, which indicates how strongly a term is related to a particular topic.

As a brief overview, Topic 2 is focused on decriminalizing sex work and ensuring safe working conditions for sex workers, many of whom are mothers. Topic 6 focuses on the bodily autonomy of women and other genders who can become pregnant. Also addressed in this topic are arguments that women, as a category, should not be defined by their ability to reproduce. Topic 11 focuses on trans rights, especially trans women, including discussions on the Gender Recognition Act, proposed extensions to the conversion therapy ban to cover trans individuals, single‐sex spaces, as well as celebrating trans joy. Topic 16 covers domestic violence and its effects on both women and children, including family court procedures. This topic also addresses cis male violence outside of the home, particularly regarding the murder of cis and trans women. Topic 18 addresses the public sexual harassment experienced by women and girls. ‘Safe’ and ‘spaces’ rank highly on this topic and refer to the need to create spaces safe from public harassment.

The issues driving the anti‐inclusion group include the UK 2021 census (Topic 1), violence and abuse (Topic 3), the trans community (Topic 7), concerns about child safeguarding (Topic 9), and the Gender Recognition Act (Topic 12). Similar to the pro‐inclusion group, the anti‐inclusion group also has a topic focused on calls to action (Topic 11).

Topic 1 focused on opposition to the addition of a gender identity question in the 2021 UK Census. Specifically, the 2021 Census was the first time that data on gender identity were gathered. As a core tenet of anti‐inclusion beliefs involves a denial or dismissal of gender identity (Pearce et al., 2020), this group protested the addition of this question with claims that it ‘erased’ the category of women by conflating ‘men’ (i.e., trans women) with cis women. Like Topic 16 for pro‐inclusion, Topic 3 also has terms such as ‘violence’ and ‘abuse’ ranked highly, however the primary focus in this topic is identifying abuse perpetrated towards the in‐group, particularly when they protest or speak out about their beliefs. Topic 7 focused on single‐sex spaces and the inclusion of trans women. Topic 9 is concerned with tweets related to ‘safeguarding’ children regarding gender affirmation practices. It positions gender affirming care as ‘experimentation’ and ‘unnecessary’ and argue that children are ‘groomed’ into trans identities. Finally, Topic 12 addresses the proposed changes to the Gender Recognition Act and concerns regarding the perceived implications for the group ‘women’, similar to Topic 1.

We then selected the topic from each group where the term ‘trans’ had the highest probability of association as shown in Tables 1 and 2 to be Topic 7 for anti‐inclusion and Topic 11 for pro‐inclusion. The resulting dataset was 4762 tweets for the anti‐inclusion group and 1465 tweets for the pro‐inclusion group. It is important to note that while the pro‐inclusion group had a distinct trans‐related topic, our close reading of the anti‐inclusion model demonstrated that most, if not all, of their topics were related to the trans community.

## CRITICAL DISCURSIVE PSYCHOLOGY

We constructed five interpretative repertoires in the trans‐related topics of each group to understand how opposing movements used victimization discourses to make sense of feminism in relation to trans women (Edley, 2001; Potter & Wetherell, 1987). Each opposing group employed two unique repertoires while the final repertoire was shared between groups.

### Pro‐inclusion repertoires

Two repertoires were used by the pro‐inclusion groups: (1) Trans women are victims and (2) Linked victimhood.

#### Trans women are victims

The first repertoire was utilized by pro‐inclusion groups to frame trans women as victims of social injustices. They drew on this repertoire primarily when making sense of current events to shape their audience's perceptions of the trans community. For instance, pro‐inclusion groups described the murder of trans girl Brianna Ghey in the UK, in February 2023, as ‘beyond harrowing’. By attaching the intensifier of ‘beyond’ to the already strong descriptor of ‘harrowing*’* (an example of extreme case formulation; Pomerantz, 1986), pro‐inclusion groups seek to persuade their audience of the severity of this murder while challenging any alternative interpretations that downplay its distressing nature. In this way, Brianna is constructed as a victim who has suffered an inexcusable injustice and is deserving of retribution and sympathy.

Pro‐inclusion groups also attempt to guide audiences to consider this event in context through the allocation of blame. Specifically, they attribute causality for Brianna's murder to the anti‐trans ideologies that ‘enabled her death to happen’, the state ‘for their murderous culture war’ on trans people, the education department for creating a ‘trans‐hostile environment’, and the media ‘for their stochastic terrorism’. By connecting Brianna's murder to these prevailing systems of transphobia, pro‐inclusion groups frame this incident as reflective of a broader, ongoing issue rather than a singular event. That is, Brianna is positioned as a victim, however, the audience is encouraged to consider all trans women as potential victims.

In this repertoire, pro‐inclusion groups appeal to audiences to grant trans women victimhood status alongside other recognized victimized populations. For instance, within prison settings, they construct trans women as a group who ‘are just as much victims as anyone else’. Here, pro‐inclusion groups respond to culturally dominant repertoires that depict trans women as predators in single‐sex women's prisons by acknowledging the vulnerability of trans women imprisoned alongside cis men (e.g., Francisco, 2021; Wilson et al., 2017). Similar claims of trans women's victimhood are made within the context of women's domestic violence refuges. Pro‐inclusion groups seek to validate trans women as victims deserving of the same concern given to others without denying the victimhood status of other marginalized groups; they acknowledge harms to cis women in these spaces as ‘horrific’ and ‘terrible’ but reallocate blame from trans women to cis men and ‘flaws in the prison system’.

#### Linked victimhood

In addition to establishing that trans women are victims, pro‐inclusion groups also drew on repertoires that connected trans women and cis women as having shared victimhood experiences. In the following two extracts pro‐inclusion groups linked trans women and cis women through a shared experience of a common perpetrator: ‘Trans people are oppressed by patriarchy. Cis women are oppressed by patriarchy’ and ‘structural oppression of women – cis and trans’.

In the first extract, repetition is used to reinforce the similarities between the two groups regarding their victimhood while across both extracts responsibility for that victimhood is allocated to a shared cause: the patriarchy. In the second extract, the specification of prefixes for the term ‘woman’ (‘cis and trans’) positions each group as a sub‐group under the superordinate category of ‘womanhood’ while maintaining their unique identities (Dovidio et al., 2010). In this way, the success of cis women in the feminist movement in overcoming patriarchal oppression is inextricably connected to trans liberation. This linked fate was further evidenced by ingroup appeals within the context of domestic violence and gender‐based violence as ‘cis & trans survivors need each other’. Here, pro‐inclusion groups assume subject positions of both dependency and solidarity with trans communities.

Pro‐inclusion groups also link the fates of both groups by portraying cis women as victims to anti‐trans rhetoric. For instance, group prototypes based on ‘passing’ (i.e., being perceived as a group member) as a cis woman were argued to harm ‘gender non‐conforming cis women most’, and cancer survivors who have ‘had a double mastectomy’. Research has reported how ideas around ‘passing’ translate into the informal policing of women's bathrooms which, in turn, can result in questioning the legitimacy of non‐prototypical cis women and can cause distress similar to what trans women experience daily (Dias et al., 2021; Hines, 2020; Watson, 2016). By invoking the personal narratives of those whose victimhood cannot be contested (i.e., cancer survivors), pro‐inclusion groups appear to emphasize the unintended consequences of transphobic policies and norms for cis women. In this way, the reality of policies affecting trans women are made relevant to all women such that it is considered in the latter's’ best interests to address transphobia as part of feminist mobilization.

The repertoire of linked victimhood was also employed in response to political engagement with cis women's rights and trans women's rights. Specifically, pro‐inclusion groups draw associations between attempts to undermine trans women's rights and the subsequent attention allocated to cis women's rights. They used lists (Wiggins, 2017) to identify and emphasize the plethora of issues affecting cis women in the United Kingdom, including ‘the dire criminal justice system, reproductive rights and the economic situation of women’ and ‘police raping and killing women’. Pro‐inclusion groups then contrasted these ingroup priorities with the current political focus on ‘unisex toilets’, ‘a minority of people's genitals’, and ‘trans women in prison’. These contrasts communicate a negative evaluation (Wiggins, 2017) of the political agenda, implying that the focus on anti‐trans policies does not resolve cis women's oppression and, instead, diverts resources and attention from areas that could improve cis women's safety and well‐being.

Together, these examples establish a repertoire that intends to persuade audiences that collaborative action between cis women and trans women is mutually beneficial for feminism. It is through an emphasis on shared victimhood and linked fate that pro‐inclusion groups attempt to mobilize the ingroup to fight for the rights of both cis women *and* trans women. Both repertoires support the stated goals of the group which are to attain ‘the liberation of all women’ and ‘to protect women, all women’ where the word ‘protect’ signals ingroup vulnerability while the quantifier ‘all’ reiterates a trans‐inclusive feminist movement.

### Anti‐inclusion repertoires

Two repertoires were used by anti‐inclusion groups: (1) trans women are perpetrators and (2) competitive victimhood.

#### Trans women are perpetrators

Anti‐inclusion groups resist the ‘trans women as victims’ repertoire created by pro‐inclusion groups. Instead, their understanding of trans women in the feminist movement is informed by a perpetrator lens. This repertoire is used to invalidate trans women as both women and victims by positioning them instead as male outgroup members who are responsible for the suffering of the ingroup.

One way in which anti‐inclusion groups invoke this repertoire is by establishing group boundary conditions based on biological sex. Consequently, trans women are categorized as having male membership, which is reinforced through deadnaming, male pronouns, and language that creates biologically‐based boundaries such as ‘male bodied person’, ‘bio males’, and ‘obvious males’. These practices serve to deny trans women their womanhood while also assigning them attributes prototypical to the male outgroup. Such attributes include possessing greater physical strength than the ingroup which is argued to create ‘a persistent climate of threat and coercion’ for cis women. In this extract, anti‐inclusion groups employ figurative language to express inescapable fear among cis women. They seek to persuade their audience that anyone with attributes that they assign as male is a potential threat to the safety and well‐being of cis women. These tactics maximize the distinctiveness between cis women and trans women while establishing the vulnerability and relative powerlessness of the ingroup.

This repertoire is also invoked during discussion of policies intended to accommodate trans women into single‐sex women's spaces. Within the prison context, these policies are claimed to put ‘vulnerable, abused women’ at further risk from ‘violent men exploiting self ID’ (i.e., ‘self identification’). This extract employs contrasts to reinforce the distinction between the two essentialist groups: the female ingroup positioned as helpless victims and the male outgroup positioned as powerful perpetrators. In the second extract, transgender identities are reduced to a strategic and temporary performance to gain access to cis women for nefarious purposes.

This constructed vulnerability is also used to underpin anti‐inclusion arguments that trans women should be excluded from single‐sex women's sports. Not only are trans women considered a threat to the physical safety of cis women, but they are also depicted as a threat to their rights. For instance, anti‐inclusion groups proffer arguments that trans women have ‘retained male advantage’ resulting in them being ‘generally stronger and faster than women’. These causal arguments frame the ingroup as too physically weak to fairly compete against trans women while reinstating boundary immutability; by ‘retaining’ the attributes associated with the male outgroup, anti‐inclusion groups contend that trans women cannot claim female group membership and therefore will always have more political and physical power than the ingroup.

#### Competitive victimhood

Contrasting the pro‐inclusion repertoire of ‘Linked victimhood’, anti‐inclusion groups understand the relationship between trans women and feminism as one of competition. They reject arguments that trans rights and cis women's rights can co‐exist: ‘The harsh reality is the choice is safety of females OR inclusion of males’. In this extract, anti‐inclusion groups seek to establish the perceived conflict between these two groups as indisputable fact. Furthermore, the capitalized contrasting device (‘OR’) emphasizes a zero‐sum relationship such that the prioritization of one group's needs (‘inclusion’) comes at the expense of another group's needs (‘safety’). The strategic comparison of ‘safety’ and ‘inclusion’ also disregards evidence that inclusive policies contribute to the safety of trans women (Wilson et al., 2017). In this way, anti‐inclusion groups attempt to persuade the audience that they are forced to choose a ‘side’ in this perceived intergroup competition.

This repertoire further positions trans women as winning this competition. Within the domain of sport, recent changes by the International Olympic Committee (2021) are argued to advantage trans women at the expense of cis women, with examples that the former ‘takes a womans winning spot, again’ and competes ‘in her female category’. These extracts depict trans women as taking something that rightfully belongs to cis women. As such, anti‐inclusion groups contend that trans women have more rights, arguing that while cis women's rights are in peril, ‘No one is “removing rights” from trans people’. The use of ‘no one’ as an example of extreme case formulation serves to invalidate and challenge trans women's claims to victimhood by implying that their rights are secure. Furthermore, reported speech (Wiggins, 2017) as a discursive device represents others' arguments (i.e., ‘removing rights’) in quotation marks to indicate scepticism. Instead, anti‐inclusion groups use minimization (Wiggins, 2017) to reduce the importance of trans women's rights by asserting that cis women's rights and well‐being are *‘*being sacrificed to validate men's feelings’. This excerpt attempt to convince audiences that cis women's rights are more legitimate than trans women's rights which are relegated to being more subjective and expendable ‘feelings’.

Strong, emotive language like ‘sacrificed’ in the previous extract is frequently employed by anti‐inclusion groups to emphasize their victimhood. For instance, they often describe their rights as being ‘demolished’, ‘stripped’, ‘stolen’, and ‘removed’. This portrayal of the ingroup as being undeservedly attacked supports subsequent justification for actions that seek to ‘defend’, ‘protect’, and ‘preserve’ *cis* women's rights, single‐sex spaces, and the very nature of their identities. They describe this intergroup competition through metaphors of battle such as being on the ‘front line of the gender war’ as well as hashtags such as #WarOnWomen. If unsuccessful, these groups contend that ‘the erosion of women's rights has genuine catastrophic, and potentially, fatal consequences’. The use of ‘genuine’ appears to increase the factuality of the claim while the use of hedging (Wiggins, 2017) in the tentative description of the consequences (‘potentially’) introduces the audience to possible futures if corrective action is not taken.

Anti‐inclusion groups also use this repertoire to make sense of the political and public responses to their collective action attempts. They assume subject positions of victims to censored speech by claiming that ‘accusations of transphobia’ are used to *‘*prevent women ever discussing areas where rights are in conflict’. Here, transphobia is minimized as a discriminatory practice and re‐conceptualized as a weapon used to silence anti‐inclusion groups. Hashtags are created to support ingroup cis women who are perceived as having been ‘punished’ and ‘silenced’ for expressing their concerns. As one example, the #IStandWithMaya hashtag refers to Maya Forstater, a cis woman in the UK who was fired from her job for in 2018 for posting her anti‐inclusion views on Twitter (Quatrini, 2022). The focus on the costs that ingroup members have received for expressing their beliefs is used to fuel their victimhood narratives by elevating the implicated individual as a metaphorical martyr for the ingroup. Indeed, ingroup members are portrayed as ‘brave’ and ‘courageous’ for expressing their beliefs.

Anti‐inclusion groups further distance themselves from being cast as perpetrators of violence by claiming that ‘women's rights are not hateful’ and that ‘for all the insults hurled at us, we do not set out to hurt’. This second extract attempts to flip the narrative to suggest that intergroup violence originates from trans‐rights proponents. Any retaliatory violence from the ingroup is implied as a justifiable reaction, but by claiming non‐violence they assume a position of moral superiority. Trans women who do critique or protest anti‐inclusion group members are portrayed as ‘gender extremists’, ‘deranged mobs’, ‘terrorists’, and a ‘gender cult’. These provocative descriptors not only intend to disqualify trans peoples' claims to victimhood by depicting them as a threat, but also heighten the seriousness of the ingroup's victimization and the urgency for audience intervention.

### Delegitimising opposition

This final repertoire is used by both groups in an attempt to delegitimise their opposition so that they can assert primary control over the victimization narrative within the feminist movement. Each group also draws on this repertoire to maximize the distinction between themselves and the opposing group. While each group draws on different discursive arguments, both intend to persuade their audience that the opposing group is not representative of feminism and is not focused on the interests and concerns of cis women.

#### Pro‐inclusion groups

Firstly, pro‐inclusion groups assert that the use of biological criteria by anti‐inclusion groups to define ‘womanhood’ categorization is patriarchal, reductionist, and can ‘harm all women who don't want to or can't reproduce’. In this way, anti‐inclusion groups are argued to ‘enforce the gender binary’ that is used ‘to suppress and control women’. These extracts not only seek to reinforce a linked fate between cis women and trans women through a shared perpetrator, but they also position anti‐inclusion groups as antithetical to feminism. Specifically, the generalization of harm to ‘all women’ portrays anti‐inclusion groups as acting inconsistently with their stated objectives, that is, contributing to the oppression of cis women.

Consequently, pro‐inclusion groups attempt to persuade their audiences that their opposition fails to understand ‘what feminism really is’; the implication being that pro‐inclusion groups, instead, possess the true definition of feminism. This definition is communicated through tweets such as: ‘True feminism is intersectional and trans inclusive’ and ‘Opposing all forms of oppression is not optional for feminists’. These extracts suggest a feminism that is expanded beyond gender identity to consider other intersecting identities as well. Furthermore, the assessment of intersectionality as ‘not optional*’* sets this behaviour as normative for feminists. As such, groups that are not mindfully intersectional or trans‐inclusive in their mobilization are denied feminist identification. Reinforcing these assessments, pro‐inclusion groups place the descriptor ‘feminist’ in quotation marks (i.e., using reported speech) when discussing opposing groups, implying disagreement with this self‐identification.

To encourage their audiences to accept this definition of feminism and reduce the discursive power of their opposition, pro‐inclusion groups employ consensus in their tweets (Wiggins, 2017). This device is used to bolster one's argument by suggesting that it is supported by others. Pro‐inclusion groups use consensus by arguing that ‘the majority of women’ are not exclusionary of trans women. As individuals often change their behaviour to act in line with ingroup norms (Turner et al., 1987), the claiming of a majority‐endorsed behaviour may be used to construct a normative position of pro‐inclusion among cis women and feminists.

Pro‐inclusion groups attempt to further delegitimise the outgroup as feminist by drawing attention to how anti‐inclusion groups ‘are openly allying’ with far‐right and conservative Christian groups that seek to undermine abortion rights. They argue that this relationship can be easily verified, thereby strengthening the trust in and veracity of their evaluation. The consequences of these coalitions are stated as ‘enabling the right's attacks on women's and wider human rights’. The vague reference to ‘wider human rights’ may attempt to elicit concern from bystander groups (i.e., those who are not cis women or feminists) and persuade them to ally against anti‐inclusion groups with the implication that ‘they could come for your rights next’.

#### Anti‐inclusion groups

Anti‐inclusion groups also consider pro‐inclusion movements as a threat to ingroup goals and draw upon a delegitimization repertoire to reduce their public, political, and discursive power. Similar to their opposition, they indicate scepticism about the feminist identification of the outgroup by prefacing ‘feminist’ descriptors with dismissive qualifiers such as: ‘actual feminists’, ‘so called feminist’, ‘self‐declared feminists’, and ‘a supposed Feminist’. Anti‐inclusion groups seek to position pro‐inclusion groups as compromising feminism by portraying them to be on the side of the perpetrator as demonstrated through the following extract: ‘Men's rights activists, sorry ‘intersectional feminists’‘. Reported speech tactics are employed to not only question the outgroup's identification but also to critique intersectionality as a core tenant of feminism.

One oppositional group member often delegitimised by anti‐inclusion groups is former First Minister of Scotland, Nicola Sturgeon. Ms. Sturgeon is a feminist who advocated for the Gender Recognition Reform Bill in Scotland with the view that ‘trans women are not the threat to women’ (Sturgeon, 2022, para. 50; see also Brooks, 2022). Anti‐inclusion groups challenge her claims to a feminist group identity through the use of quotation marks around the ‘feminist’ descriptor and instead re‐position Ms. Sturgeon as a perpetrator to the ingroup by creating the hashtag #SturgeonDestroyerOfWomensRights.

By including trans women into the boundaries of womanhood, pro‐inclusion cis women like Ms. Sturgeon are constructed as ingroup traitors by having ‘sold out women’. In this way, they are framed as contributing to the ongoing oppression of cis women while advancing the interests of the perceived perpetrators (i.e., trans women). Anti‐inclusion groups lament ‘the huge number of feminists who kowtow to men’ and who ‘bend the knee to gender ideology’. In these extracts, pro‐inclusion feminists are depicted as submissive and lacking autonomy, which are reminiscent of gender stereotypes (Ellemers, 2018). The vague reference to the quantity of these easily influenced pro‐inclusion feminists (‘huge number’) positions the ingroup as the exception, as impervious to the power and manipulation of ‘men’ and therefore reiterates their *‘*brave’ attributes. Through this delegitimization of their opposition, anti‐inclusion groups seek to persuade their audiences that their version of feminism is the only one that works in the best interests of cis women. They frame feminism as being ‘about females’ and for ‘all of the women and girls, and us only’ where the generalizing ‘all’ is employed differently than pro‐inclusion groups to claim exclusion based on biological definitions of womanhood.

## DISCUSSION

The current research aimed to examine how opposing groups construct feminism in relation to trans women using victimization discourses. By combining automated text analytic methods with in‐depth qualitative analysis, we found that both groups endorsed identities of collective victimization (Bar‐Tal et al., 2009; Vollhardt, 2020), albeit in different ways and for different purposes. These intergroup distinctions can be captured by Vollhardt's (2015) dual categorization of collective victimhood into exclusive victim consciousness and inclusive victim consciousness. For instance, anti‐inclusion groups constructed a feminist victimhood that was determined by biological characteristics and gender essentialism (Morgenroth & Ryan, 2021), thereby excluding trans women. They considered themselves to be in direct competition with trans women and emphasized the primacy of ingroup suffering and oppression as compared to this outgroup. Such behaviour is consistent with the adoption of an exclusive victim consciousness (Vollhardt, 2015).

Anti‐inclusion groups minimized trans women's victimhood and instead portrayed them as perpetrators whose rights came at the expense of cis women. These zero‐sum beliefs are used to drive a narrative of unfairness (Noor et al., 2008; Norton & Sommers, 2011) and concern about the future vitality of their group identity (Belavadi & Hogg, 2023; Solomon & Martin, 2019) that attempts to mobilize ingroup members towards collective action. That is, anti‐inclusion groups express concern that the category and meaning of ‘woman’ is being ‘erased’ through the development of a superordinate group that includes trans women. These flexible group boundaries are perceived to threaten ingroup identities and their distinctiveness from outgroups which, in turn, provokes protective action (Jetten et al., 1997; Wohl et al., 2011). Consequently, anti‐inclusion groups seek to reinforce the distinctiveness between the ingroup and trans women by controlling victim/perpetrator narratives and emphasizing the comparative disadvantage experienced by the ingroup, demonstrating a victimhood that is competitive in nature (Noor et al., 2012).

To strengthen their claims to victimhood, anti‐inclusion groups also distanced themselves from the perpetrator identity by denying ingroup violence towards the trans community. This denial discourse (Kreis, 2017; van Dijk, 1992) is commonly used within racist talk to maintain positive ingroup‐presentation while disparaging the outgroup. For instance, Solomon and Martin (2019) found that Blue Lives Matter groups challenged depictions of ingroup members (i.e., police officers) as perpetrators of violence towards the Black community by portraying their opposition as the instigators of violence and the ingroup's actions as subsequently justifiable responses. Thus, the ingroup is constructed as not only morally superior to their opposition, but also as the sole victims (Noor et al., 2008). This attempt to maximize intergroup differences along victim/perpetrator axes when victimhood is incorporated into the ingroup identity is consistent with social identity theorizing, particularly when victimization is considered a valued identity (Tajfel & Turner, 1979). Additionally, anti‐inclusion groups dismissed accusations of transphobia as impingements on their free speech. Claims of being silenced by a marginalized group are often leveraged by advantaged groups to assert their victimhood and diminish ingroup wrongdoing (Danbold et al., 2022).

While anti‐inclusion groups constructed a victimhood based on competition (Noor et al., 2012), pro‐inclusion groups emphasized similarities between the oppression of cis women and trans women, consistent with an inclusive victim consciousness (Vollhardt, 2015). By acknowledging trans women as victims to cis male and patriarchal violence, pro‐inclusion groups recategorised trans women and cis women under a superordinate identity of shared oppression (Gaertner et al., 1993). For this reason, other researchers have previously identified trans activism and feminism as complementary movements rather than rivals (Platero & Ortega‐Arjonilla, 2016; Williams, 2016). The data presented in the current work provide evidence of these re‐categorization processes conducted by pro‐inclusion groups.

These inclusive victim ingroup boundaries were also extended beyond trans women to construct an intersectional movement. Intersectionality considers the ways in which the various social identities that individuals simultaneously inhabit interact with each other and are differentially located to power and privilege (Cole, 2009; Crenshaw, 1991). Feminism is often recognized as an intersectional movement: a survey of attendees at the 2017 Women's March in Washington, USA, documented that women's rights advocates were motivated by a range of issues, demonstrating intersectionality in their mobilization (Fisher et al., 2017). This is contrasted with the lack of anti‐inclusion focus on, and sometimes derision of, intersectionality. These flexible boundaries developed by the pro‐inclusion group help to create the most inclusive ingroup, expanding the reach of the movement and encouraging bystanders to participate which, in turn, increases the chance of the movement's success (Reicher & Hopkins, 1996). Feminists have identified how expansive definitions of womanhood are needed to build ‘strength in numbers’ (Serano, 2013, p. 111; Watson, 2016).

The function of these inclusive boundaries is to create a sense of linked fate (Dawson, 1994) among ingroup members such that the suffering of one group has direct consequences for the suffering of the other. In turn, this linked fate encourages cis women's acceptance of and advocacy for trans women as they jointly struggle against a common perpetrator (Craig & Richeson, 2012). Indeed, perceiving shared fate and adopting an inclusive victimhood consciousness more generally has been linked to collective and political action (Subašić et al., 2011; Vollhardt, 2015; Yildiz & Verkuyten, 2011). These findings reiterate suggestions from previous work that an inclusive victim consciousness can be promoted by highlighting shared suffering (Vollhardt, 2015).

These results speak to two distinct feminisms fashioned by opposing movements. These differences can be observed not only in the way that they constructed victimization, but in their mobilization focus as well; our topic models showed that while both groups considered themselves victimized across a variety of issues, there was minimal overlap between the two models. For instance, pro‐inclusion groups focused on a variety of issues impacting cis women, trans women, and others adversely affected by patriarchal structures such as sex work, reproductive rights, and public sexual harassment. These dimensions of victimization are considered typical to feminist movements (Morgan, 1996; Shaw, 2023). The diversity of issues reflected in the pro‐inclusion topic model is also highlighted when compared to the anti‐inclusion topic structure; despite having three‐times fewer tweets than the anti‐inclusion group, the pro‐inclusion model yielded a larger number of topics indicating a wider movement focus.

The topics for the anti‐inclusion groups, however, depict a movement that is largely trans‐focused. Close reading indicates a single‐issue movement that is driven by an anti‐trans agenda rather than pro‐woman motivations. That is, while anti‐inclusion groups did, to some extent, discuss typical topics for feminist mobilization this discussion occurred at a far lower rate than anti‐trans issues which emerged as the most prevalent topics. These observations support work from Morgenroth et al. (2022) who find that individuals who express opposition to inclusive policies are driven more by negative attitudes towards the trans community than concerns about male violence towards cis women.

Furthermore, at times the anti‐inclusion group's conceptualization of a worthy victim was restricted to cis women who shared their beliefs (i.e., ingroup members). For instance, while both groups addressed issues of ‘abuse’ and ‘violence’ (topic 16 for pro‐inclusion and topic 3 for anti‐inclusion), these terms were contextualized as domestic violence for pro‐inclusion groups and perceived ingroup violence for anti‐inclusion groups when they spoke out about their beliefs on trans women's inclusion. These restricted boundaries are contrasted with the pro‐inclusion movement which sought to create an inclusive ingroup (Reicher & Hopkins, 1996). Together, our findings provide support for arguments that anti‐inclusion perspectives are not just a fringe group of feminism but might not actually represent feminist principles at all (e.g., Bailey, 2022; Rogers, 2024).

Despite these differences, both groups positioned themselves as representing true feminism while questioning the legitimacy of their opposition. These strategies are consistent with the prototypicality dimension of the social identity approach to leadership (Haslam et al., 2020; Reicher et al., 2005). This approach argues that leaders are the most influential when they can successfully establish themselves as being representative of the group. Previous research has documented these processes online by showing how social media accounts can emerge as prototypical movement leaders that can influence social action participation (Uysal et al., 2021). As such, the extent to which each feminist group can convince their audiences that they reflect true feminism may determine their ability to claim primacy over the victimization narrative and successfully mobilize for their objectives. These results indicate that each group is aware of the other and views them a threat to the ingroup, underlining the importance of research that considers opposing movements together (Thomas & Osborne, 2022).

### Theoretical implications

The current study has contributed to the growing literature on the communication strategies of opposing social movements and expands it to a new context. Specifically, we identify both differences and commonalities (Thomas & Osborne, 2022) in the ways that opposing feminist groups craft their movement messages to construct group boundaries, define their identities and those of the broader feminist movement, and mobilize their audiences. However, our study also innovatively expands upon the existing opposing movements literature by examining competitive sub‐groups within a superordinate group (the feminist movement). Unlike previous studies that typically focus on differences *between* movements (such as the Black Lives Matter and Blue Lives Matter or All Lives Matter movements) and assume homogeneity *within* movements, our analysis reflects the complexities and nuances of the internal dynamics within diverse social movement structures. Additionally, our research aligns with Brown et al. (2022) to show the usefulness of big data approaches to examine and compare opposing movements online.

The results of the current study also support Solomon and Martin's (2019) assertion that collective victimization provides a meaningful framework for understanding social movements in conflict. When applied to social movements, victimization narratives offer explanations and a sense of purpose and direction for groups who perceive themselves as victims of injustices. Our research shows how victimhood narratives are central to how anti‐inclusion and pro‐inclusion groups positioned themselves and their opposition to their followers and audiences. Victimization as examined in the current research also aligns with and contributes to existing social psychology literature that finds perceived injustice as predictive of collective action (e.g., Acar & Uluğ, 2016; van Zomeren et al., 2008; Walker & Smith, 2002). As such, our research presents another way to think about the content of perceived injustice and the actual strategies that opposing movements to mobilize in response.

We expand on Solomon and Martin (2019) application of competitive victimhood to consider different types of victim consciousness within the broader collective vicitmization framework. Using Vollhardt's (2015) conceptualization, the current work showed that conservative groups (i.e., anti‐inclusion) adopted an exclusive victim consciousness while progressive groups (pro‐inclusion) adopted an inclusive victim consciousness. Importantly, our research demonstrated for the first time how these narratives of victimization were discursively constructed and contested through the examination of each groups' social media communication.

### Limitations and future directions

To begin, the disagreement among scholars about how to best determine *k* (i.e., the optimal number of topics when conducting topic modelling) means that our dataset, when assessed using with other topic‐calculation methods, could potentially result resulted in different topic formulations which, in turn, may have altered interpretation of the dataset. Our approach was guided by best practices within the limitations of existing knowledge, acknowledging that a ‘perfect fit’ of *k* does not exist and is instead optimized by considering research objectives and the coherence of the model (Weston et al., 2023). However, the strength of the fit for our topic model may have been further empirically supported by inter‐rater agreements on *k* (such as through the use of Cohen's *K*; Cohen, 1960).

Furthermore, the data used in the current research are not intended to represent all feminist perspectives on this issue within or outside of the United Kingdom. While efforts were made to ensure a comprehensive sampling strategy using varied search and selection tools, it is likely some perspectives are missing either due to being overlooked or because of account privatization. Furthermore, although many contemporary social movements mobilize on Twitter, our approach is limited as the demographics of Twitter users are not representative of the broader society (Boyd & Crawford, 2012; Duggan, 2015; Wojcik & Hughes, 2019). Thus, despite the examination of Twitter data lending to the ecological validity of the current approach – given that social movements commonly mobilize and organize online – caution should be exercised when generalizing these findings beyond the groups studied.

In addition, we have argued that social movements strategically position themselves and others using victimization discourses to achieve certain objectives, such as eliciting support from bystanders, and motivating mobilization among ingroup members. Our current research is unable to ascertain the effectiveness of these discursive attempts on the intended (and unintended) audiences. Future research should seek to understand how opposing movements' communication strategies are received by various audiences, such as ingroup members (i.e., followers), outgroup members, and bystanders. Similarly, engagement rate analytics using available public metrics (such as the degree of likes, retweets, comments on a post relative to a user's follower count; X Corp., 2024) may provide an additional measure of the effectiveness of mobilization communication between groups and across different types of posts (i.e., posts that contain different types of victimhood language).

## CONCLUSION

The present research used naturalistic, real‐world data examine the victimization discourses constructed by opposing ideological groups within the feminist movement regarding the inclusion of trans women. Our research provides novel insights into the social movement literature and recognizes the important functions of victimhood in movement messaging, particularly among conflicting groups. We also show how topic modelling, as an emerging technique in social psychology, can be combined with qualitative approaches to generate multi‐levelled insights about social media data. As social media is increasingly becoming a key part of how movements mobilize, these methods provide an important avenue to document how social change occurs.

## AUTHOR CONTRIBUTIONS

**Christina Maxwell:** Conceptualization; methodology; writing – original draft; writing – review and editing; project administration. **Hema Preya Selvanathan:** Conceptualization; methodology; supervision; writing – review and editing. **Sam Hames:** Conceptualization; methodology; supervision; writing – review and editing. **Charlie R. Crimston:** Conceptualization; supervision; writing – review and editing. **Jolanda Jetten:** Conceptualization; supervision; writing – review and editing.

## CONFLICT OF INTEREST STATEMENT

There are no financial sponsorships or conflicts of interest to declare for the current work.

## ETHICS STATEMENT

This study received ethics approval from the University of Queensland Ethics Committee (2022/HE002331).

## Supporting information


Data S1.

## Data Availability

The de‐identified dataset and analytic scripts can be accessed at https://osf.io/4nmjr/?view_only=bb2e96ef12074f909cac8fed4681eebc

## Footnotes

^1^

https://followerwonk.com/bio
