# Supplementary material for: A mixed‐methods approach to understand victimization discourses by opposing feminist sub‐groups on social media
Source: Br J Soc Psychol. 2024 Jul 5;64(1):e12785. doi: 10.1111/bjso.12785 (PMC11600392; doi:10.1111/bjso.12785)
Supplement: Supplementary file 1 — Data S1. [file BJSO-64-0-s001.docx]

**Supplemental Material**

Table S1. Data Description

| **Account** | **Range of Followers** | **Year Account Created** | **Range of Tweets** |
| --- | --- | --- | --- |
| Pro |  |  |  |
| 1 | 1,000 - 5,000 | 2011 | 1,000 - 5,000 |
| 2 | 1,000 - 5,000 | 2008 | 1,000 - 5,000 |
| 3 | 5,000 - 10,000 | 2010 | 5,000 - 10,000 |
| 4 | 1,000 - 5,000 | 2016 | 1,000 - 5,000 |
| 5 | 1,000 - 5,000 | 2021 | 1,000 - 5,000 |
| 6 | 5,000 - 10,000 | 2010 | 5,000 - 10,000 |
| 7 | 5,000 - 10,000 | 2019 | 1,000 - 5,000 |
| 8 | 10,000 - 50,000 | 2010 | 5,000 - 10,000 |
| 9 | 1,000 - 5,000 | 2019 | 1,000 - 5,000 |
| 10 | 10,000 - 50,000 | 2017 | 25,000 - 50,000 |
| 11 | 50,000 - 75,000 | 2014 | 5,000 - 10,000 |
| 12 | 10,000 - 50,000 | 2022 | 5,000 - 10,000 |
| 13 | 0 - 1,000 | 2012 | 0 - 1,000 |
| 14 | 10,000 - 50,000 | 2018 | 1,000 - 5,000 |
| Anti |  |  |  |
| 1 | 1,000 - 5,000 | 2021 | 10,000 - 25,000 |
| 2 | 10,000 - 50,000 | 2018 | 50,000 - 75,000 |
| 3 | 1,000 - 5,000 | 2021 | 1,000 - 5,000 |
| 4 | 1,000 - 5,000 | 2018 | 5,000 - 10,000 |
| 5 | 5,000 - 10,000 | 2011 | 10,000 - 25,000 |
| 6 | 1,000 - 5,000 | 2021 | 0 - 1,000 |
| 7 | 5,000 - 10,000 | 2021 | 5,000 - 10,000 |
| 8 | 5,000 - 10,000 | 2018 | 1,000 - 5,000 |
| 9 | 50,000 - 75,000 | 2017 | 25,000 - 50,000 |
| 10 | 10,000 - 25,000 | 2014 | 25,000 - 50,000 |
| 11 | 5,000 - 10,000 | 2018 | 1,000 - 5,000 |
| 12 | 50,000 - 75,000 | 2016 | 10,000 - 25,000 |
| 13 | 50,000 - 75,000 | 2021 | 10,000 - 25,000 |

*Note.* The data description represents these accounts at the time of data collection

**Data Collection Additional Information**

Following the sampling strategy outlined in the main manuscript, we collected an initial sample size of 88 accounts (39 pro-inclusion and 49 anti-inclusion). We applied our eligibility criteria (as detailed in main manuscript) to all accounts located through the sampling strategy to result in the 27 accounts used for analysis (13 anti-inclusion accounts and 14 pro-inclusion accounts).

**Dataset Pre-Processing for Topic Modelling Additional Information**

In addition to the steps outlined in the main manuscript that are typical in pre-processing datasets for LDA topic modelling, we made some additional changes that were specific to our research aims and questions. Firstly, we removed quotations from our datasets (i.e., anything appearing within “”). When selecting our data sources, we observed that a high frequency of anti-inclusion accounts posted tweets that only contained quoted content from other sources (e.g., news articles, blogs, other users). As these quotes were often shared among similar accounts, we believed this could bias the resulting topic model. We were also more interested in how each group spoke about these topics in their own words rather than echoing someone else’s perspective.

We also retained emojis by converting into Unicode and then retrieving their Common Locale Data Repository (CLDR) descriptive names. Emojis are integral to how people communicate tone and emotion on social media (Wagner et al., 2020). Within the current context in particular, emojis are used as a shorthand for opposing feminist groups to indicate their beliefs to others. For instance, anti-inclusion groups tend to use a series of green, white, and purple hearts to signal their group membership. These colours are reminiscent of the Suffragette Movement but have been recently co-opted by gender critical feminists to indicate exclusionary beliefs towards trans women, Alternatively, pro-inclusion groups may use the transgender flag to signify an inclusion of trans women within their movements. In this way, emojis are used by both groups to signal to others their ingroup’s beliefs about inclusion and the boundaries of who is and is not granted membership into the group ‘women’.

We also elected to avoid stemming and lemmatising (reducing a word to its root word) as these were considered meaningful in the current dataset (Brookes & McEnery, 2018). For instance, to delegitimise the gender identity of transgender individuals, anti-inclusion groups often use outdated and offensive language such as "transgenders" or "transgendered". We also created a custom stop word list that removed references to twitter urls (“amp”).

As pre-processing decisions can affect the results of the topic model (Doogan et al., 2023), we conducted several LDA topic models with varying pre-processing steps (e.g., with and without hashtags, emojis, or lemmatisation) and observed the results. Our final selection was based on those models that enhanced interpretability and addressed our research aims. The code used to perform this pre-processing can be located in the accompanying OSF file: <https://osf.io/4nmjr/?view_only=bb2e96ef12074f909cac8fed4681eebc> .

References

Brookes, G., & McEnery, T. (2018). The utility of topic modelling for discourse studies: A critical evaluation. *Discourse Studies, 21*(1), 3–21. https://doi.org/10.1177/1461445618814032

Doogan, C., Buntine, W., & Linger, H. (2023). A systematic review of the use of topic models for short text social media analysis. *Artificial Intelligence Review*, 1–33. <https://doi.org/10.1007/S10462-023-10471-X>

Wagner, A., Marusek, S., & Yu, W. (2020). Emojis and law: Contextualized flexibility of meaning in cyber communication. *Social Semiotics*, *30*(3), 396-414. https://doi.org/10.1080/10350330.2020.1731198
